# Novel Polyarylene Ether Nitrile/BaTiO_3_-Decorated WS_2_ Nanohybrid Crosslinked Nanocomposites for Thermal-Stable Dielectrics

**DOI:** 10.3390/polym18060747

**Published:** 2026-03-19

**Authors:** Xiaohua Mao, Jingtao Zhou, Junda Wu, Siyi Chen, Pan Wang, Yong You

**Affiliations:** 1 Key Laboratory of Pollution Control Chemistry and Environmental Functional Materials for Qinghai-Tibet Plateau of the National Ethnic Affairs Commission, School of Chemistry and Environment, Southwest Minzu University, Chengdu 610041, China; 2 School of Mechanical Engineering, Chengdu University, Chengdu 610106, China

**Keywords:** polyarylene ether nitrile, nanocomposites, crosslinking, dielectric films

## Abstract

To prepare high-temperature-resistant dielectric composite films, a novel three-dimensional nanofiller was fabricated using carboxylated polyarylene ether nitrile as a bridge, which tightly loads BaTiO_3_ nanoparticles onto WS_2_ nanosheets (WS_2_@BT) via in situ chemical bonding. Afterward, the WS_2_@BT nanofiller was introduced into the polyarylene ether nitrile (PEN) matrix, and high-temperature heat treatment was performed to form a crosslinked network, yielding CPEN/WS_2_@BT nanocomposites. Notably, the modified WS_2_@BT effectively improves the compatibility between the nanoparticles and the PEN matrix, which is superior to the compatibility of unmodified nanofillers with the matrix. Moreover, after crosslinking, CPEN/WS_2_@BT exhibits excellent comprehensive performance: when the filler content is 30 wt%, its glass transition temperature (*T*_g_) reaches 257.83 °C, significantly higher than that of PEN/WS_2_@BT, and its dielectric constant is 193% higher than that of pure CPEN. In addition, the dielectric temperature coefficient remains below 1 × 10^−3^ °C^−1^ in the range of 25–220 °C. Overall, this work provides an effective and reliable strategy for preparing high-performance, high-temperature-resistant composite dielectric films.

## 1. Introduction

As global attention gradually shifts toward energy and ecological issues, renewable energy, particularly electrical energy, has attracted considerable attention. Meanwhile, advancements in science and technology have driven the rapid development of the electronic industry, which has been accompanied by increasingly stringent requirements for energy storage technologies. High-density energy storage capacitors play an indispensable role in various fields, including aerospace, electrical engineering, and microelectronics [1,2]. Furthermore, the research direction of electronic devices has evolved from merely improving energy storage capacity to developing novel electronic devices that are lightweight, low-cost, easily processable, and endowed with high-density energy storage capabilities. However, conventional dielectric materials, such as inorganic ceramics, suffer from inherent limitations, including brittleness, restricted mechanical properties, and harsh preparation conditions [3,4]. In contrast, polymer dielectric materials are lightweight, flexible, and hold great potential for applications in film capacitors [5,6]. Nevertheless, the low dielectric constant limits their utilization in scenarios requiring high dielectric performance [7,8]. To overcome the limitations of both polymer and ceramic materials, researchers have explored the incorporation of high-permittivity inorganic nanomaterials into polymer matrices to fabricate polymer nanocomposite dielectric materials with enhanced permittivity [9]. Nanocomposites provide an effective solution to the aforementioned material drawbacks, integrating the advantages of both types of dielectric materials [10,11,12,13,14]. However, nanocomposites still suffer from several critical challenges. In particular, the poor compatibility between inorganic ceramics and polymer matrices, combined with the high surface energy of nano-ceramic fillers, tends to induce severe agglomeration under high filler loading, thereby resulting in elevated dielectric loss. Surface modification methods, including organic molecule grafting [15,16,17] and surface group functionalization [18], can effectively mitigate this issue. Therefore, selecting new functional nanomaterials that enable high dielectric constant and low dielectric loss at low filler loadings has become an effective strategy for advancing dielectric material performance.

Polyarylene ether nitrile (PEN) is a widely used polymer for the fabrication of dielectric composites [19]. It exhibits excellent comprehensive properties, including high-temperature resistance, chemical stability, insulation, and mechanical strength [20,21,22,23]. Notably, PEN contains strongly polar nitrile groups on the side chains of its aromatic rings, which enable strong interactions with other polar groups. However, its low dielectric constant restricts its application scope. Thus, enhancing the dielectric constant of PEN while maintaining low dielectric loss has emerged as a key research focus [24]. Barium titanate (BaTiO_3_, BT) is a commonly used dielectric ceramic material, characterized by its high dielectric constant and low dielectric loss [25]. Two-dimensional transition metal sulfide compounds are a class of semiconductor materials with unique band gap structures and outstanding electrical, optical, and mechanical properties. These materials possess tunable conductivity and excellent flexibility, making them promising candidates for improving the dielectric constant and reducing the dielectric loss of embedded capacitive materials [26,27,28]. Tungsten disulfide (WS_2_), a typical two-dimensional transition metal sulfide, features an appropriately sized band gap and high carrier mobility, rendering it suitable as a channel material in electronic devices. Compared with conventional BT filled composites that often suffer from nanoparticle aggregation and WS_2_-based systems with limited dielectric enhancement, the WS_2_@BT hybrid architecture integrates the high permittivity of BT with the large surface area of WS_2_ nanosheets, generating abundant heterogeneous interfaces and enhanced interfacial polarization. This hierarchical structure is beneficial for improving dielectric response and structural stability of the composites [8].

In this work, to obtain composite dielectric materials with high dielectric constant, low dielectric loss, excellent heat resistance, and processability, carboxylated polyarylene ether nitrile (PEN-COOH) was employed as a “bridge” to connect high-dielectric-constant BT nanoparticles and two-dimensional lamellar WS_2_ nanosheets. Specifically, surface modification of BT and WS_2_ was first carried out using PEN-COOH as the grafting agent, which not only enhanced the compatibility between the nanofillers and the polymer matrix but also reduced the surface energy of the nanofillers, thereby suppressing filler agglomeration and constructing a three-dimensional nanofiller network structure. Subsequently, the modified BT and WS_2_ nanofillers were uniformly incorporated into the polymer matrix to fabricate composite dielectric films. Finally, the as-prepared composite dielectric films were subjected to high-temperature heat treatment to further optimize their structure and improve their thermal stability. On this basis, the dielectric properties, thermal stability, and mechanical properties of the composite dielectric films were systematically investigated, with a focus on exploring their potential applications in the field of high-temperature dielectric materials.

## 2. Experimental Methods

### 2.1. Materials

Barium titanate (BaTiO_3_, BT, ~100 nm, 99.9%), sodium hydroxide (NaOH, AR), and tetrahydrofuran (THF, AR) were obtained from Shanghai Aladdin Biochemical Technology Co. Ltd. (Shanghai, China). Tungsten disulfide (WS_2_, 99.8%) was purchased from Shanghai Alfa Aesar Chemicals Co. Ltd. (Shanghai, China). Hydrogen peroxide (H_2_O_2_, 30%) was obtained from Kelong Reagent in Chengdu, China. N-methylpyrrolidone (NMP, AR) was purchased from Shanghai Titan Scientific Co., Ltd. (Shanghai, China). None of the aforementioned materials underwent further purification. The PEN was synthesized in the laboratory.

### 2.2. Preparation of WS_2_@BT Nanoparticles

WS_2_ nanosheets were exfoliated via a ball-milling technique. Subsequently, 4 g of WS_2_ powder was added to 100 mL of a 2 M NaOH solution. The mixture was mechanically stirred continuously at 300 rpm for 2 h at room temperature, accompanied by intermittent ultrasonic treatment. The sonicated WS_2_ dispersion was then magnetically stirred at room temperature for 24 h. After being washed repeatedly with deionized water until neutral, the dispersion was dried at 80 °C for 12 h to obtain hydroxyl-functionalized WS_2_ (WS_2_-OH) nanoparticles. For the preparation of hydroxyl-functionalized BaTiO_3_ (BT-OH) nanoparticles, 4 g of BT nanoparticles was dispersed in 50 mL of H_2_O_2_ solution and refluxed for 4 h. After washing with deionized water several times, the BT-OH nanoparticles were dried at 80 °C for 12 h.

**Figure 1:**
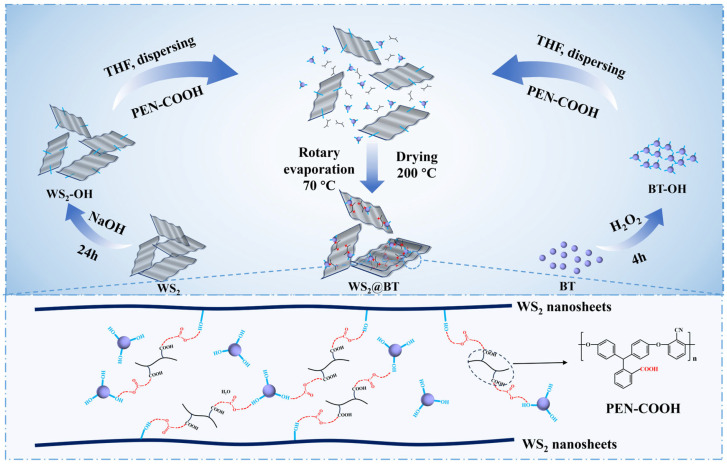
Schematic illustration of the preparation process for WS_2_@BT nanohybrids.

Surface modification of the as-prepared WS_2_-OH and BT-OH particles was performed using PEN-COOH as the grafting agent. 2 g of WS_2_-OH and 1 g of BT-OH were dispersed in 30 mL of tetrahydrofuran and sonicated for 1 h. The dispersion was then transferred to an electric heating device and heated under continuous stirring. Meanwhile, 0.3 g of PEN-COOH was dissolved in 10 mL of tetrahydrofuran, and the solution was slowly added into the above dispersion. The mixture was heated and stirred continuously in the electric heating device for 30 min, followed by rotary evaporation at 70 °C to remove the solvent. The dried sample was collected and further heated at 200 °C for 4 h. Finally, the resulting product (denoted as WS_2_@BT) was washed with ethanol and deionized water several times, dried at 80 °C for 6 h, and collected for further use. The corresponding preparation process is shown in Figure 1.

### 2.3. Preparation of PEN/WS_2_@BT Nanocomposite Films

The PEN used in this study was laboratory-synthesized [20]. To achieve uniform dispersion, WS_2_@BT was mixed with a given amount of NMP and sonicated for 30 min. A precise amount of PEN powder was then added into the mixture, followed by heating and stirring for 30 min. The obtained solution was cast onto glass substrates to form precursor films, which were sequentially dried at 80 °C, 100 °C, 120 °C, and 160 °C for 1 h, and at 200 °C for 2 h. Through this procedure, a series of PEN/WS_2_@BT nanocomposite films with WS_2_@BT loadings of 0 wt%, 5 wt%, 10 wt%, 20 wt%, and 30 wt% were fabricated, denoted as PEN, PEN/WS_2_@BT 5, PEN/WS_2_@BT 10, PEN/WS_2_@BT 20, and PEN/WS_2_@BT 30, respectively. For comparison, nanocomposite films filled with unmodified WS_2_/BT mixtures at loadings of 5 wt%, 10 wt%, 20 wt%, and 30 wt% were also prepared via the same procedure, designated as PEN/WS_2_-BT 5, PEN/WS_2_-BT 10, PEN/WS_2_-BT 20, and PEN/WS_2_-BT 30, respectively. Finally, all nanocomposite films were subjected to high-temperature heat treatment at 320 °C for 4 h and 340 °C for 4 h to achieve chemical crosslinking between the cyano groups. The resulting crosslinked films were labeled as CPEN, CPEN/WS_2_@BT 5, CPEN/WS_2_@BT 10, CPEN/WS_2_@BT 20, CPEN/WS_2_@BT 30 respectively.

### 2.4. Characterization

Fourier transform infrared (FTIR) spectra of all samples were recorded using an IR spectrophotometer (IR200, Thermo Nicolet, Waltham, MA, USA) in the wavenumber range of 400–4000 cm^−1^. The surface composition of samples was determined through X-ray photoelectron spectroscopy (XPS, ESCA-2000 Multilab apparatus, VG Microtech, Sussex, UK) using an Al-Ka excitation. The zeta potential of the nanoparticles was measured with a Malvern ZEN3690/MPT-2 instrument. The morphologies of exfoliated WS_2_ nanosheets were observed via a transmission electron microscope (TEM, JEOL-JEM-F20, Tokyo, Japan) operated at an accelerating voltage of 200 kV. Scanning electron microscopy (SEM, JEOL-JSM-7500, Nippon Electronics Co., Ltd., Tokyo, Japan) was employed to characterize the nanoparticles and cross-sectional morphologies of the nanocomposite films. X-ray diffraction (XRD) analysis was performed on an X-ray diffractometer (XD-6, Beijing Pu-Analysis General Instrument Co., Ltd., Beijing, China) using Cu Kα radiation (λ = 1.5406 Å), with a 2θ range of 5–85°. The surface chemical composition of the samples was analyzed by X-ray photoelectron spectroscopy (XPS) using an ESCA-2000 Multilab apparatus (VG Microtech) with Al Kα excitation. Dielectric properties were tested using a digital LCR bridge (TH2819A, TongHui Electronics, Dongguan, China) over a frequency range of 100 Hz to 1 MHz; prior to measurement, conductive silver paste was uniformly coated on both sides of the films to ensure good electrical contact. Thermal gravimetric analysis (TGA) was carried out on a thermogravimetric analyzer (TA Q-50, TA Instruments, New Castle, DE, USA) under a nitrogen atmosphere, with a temperature range of 50–800 °C and a heating rate of 20 °C/min. The mechanical properties of the nanocomposite films were measured using a universal testing machine (QX-W200, Shanghai Qixiang Testing Instrument Co., Ltd., Shanghai, China) at a strain rate of 5 mm/min. Differential scanning calorimetry (DSC, TA Q-2000, TA Instruments, New Castle, DE, USA) was used to evaluate the thermal properties of the films at a heating rate of 10 °C/min. Additionally, the heat transfer properties of the nanocomposite films were characterized by infrared thermography (E64501, FLIR, Wilsonville, OR, USA).

## 3. Results and Discussion

### 3.1. Characterization of WS_2_@BT Nanofilles

**Figure 2:**
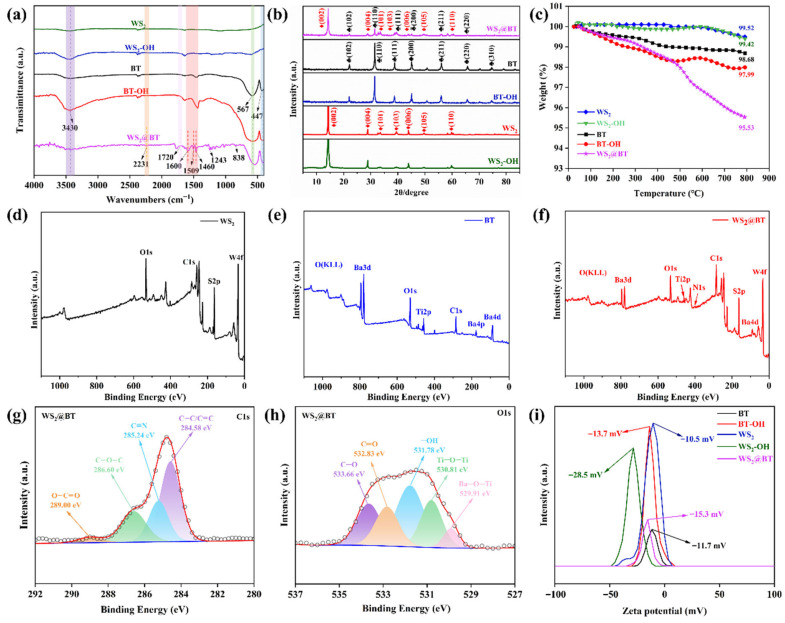
Characterization of nanofillers: (**a**) FTIR spectra; (**b**) XRD patterns; (**c**) TGA curves; (**d**–**f**) XPS survey spectra; (**g**) C1s, (**h**) O1s XPS spectra of nanofillers; (**i**) zeta potential spectra.

To confirm the surface modification effect of the fillers, chemical structure characterization was performed, which are shown in Figure 2. The FTIR spectra of the fillers are presented in Figure 2a. It can be observed that the absorption peaks at 567 cm^−1^ and 447 cm^−1^ in both WS_2_@BT and pure BT correspond to the vibrational absorption peaks of Ti-O bonds [29]. The absorption peak at 3430 cm^−1^ is attributed to the -OH groups. In WS_2_@BT, the absorption peaks at 1460 cm^−1^, 1481 cm^−1^, and 1588 cm^−1^ are assigned to the stretching vibrational absorption peaks of the benzene ring, while the absorption peak at 838 cm^−1^ corresponds to the bending vibrational absorption peak of the benzene ring [30]. Additionally, the asymmetric stretching vibrational absorption peak of the aryl ether bond in WS_2_@BT appears at 1243 cm^−1^, and an absorption peak at 2231 cm^−1^ is observed, which is the stretching vibrational absorption peak of the nitrile group (-C≡N) in WS_2_@BT [31]. This indicates that the surface of WS_2_@BT is covered with nitrile functional groups. Notably, an absorption peak of the ester bond at 1750 cm^−1^ not observed in the FTIR spectrum of WS_2_@BT [32]. This phenomenon is attributed to the chemical reaction between the carboxyl groups on the PEN molecular chain and the hydroxyl groups on the filler surface, leading to the formation of ester bonds, thereby confirming the successful esterification of WS_2_-OH and BT-OH with PEN-COOH.

The crystal structures of various nanoparticles were characterized by X-ray diffraction (XRD), and the corresponding patterns are presented in Figure 2b. For pure BT, characteristic diffraction peaks are observed at 2θ = 22.4°, 31.8°, 39.1°, 45.4°, 56.4°, 66°, and 75°, corresponding to the (100), (110), (111), (200), (211), (220), and (310) crystal planes, respectively. Pure WS_2_ exhibits diffraction peaks at 2θ = 14.3°, 28.9°, 33.5°, 39.5°, 43.9°, 49.6° and 58.4°, assigned to the (002), (004), (101), (103), (006), (105) and (110) crystal planes. For WS_2_@BT, all characteristic peaks of BT and WS_2_ are clearly detected at their respective positions with almost unchanged relative intensities. These results confirm the successful preparation of WS_2_@BT nanofillers, and the surface grafting process does not alter the intrinsic crystalline structures of BT and WS_2_.

To further determine the surface grafting amount of the fillers, thermogravimetric analysis (TGA) was conducted under a nitrogen atmosphere, and the TGA curves of the fillers are displayed in Figure 2c. The mass losses of WS_2_, BT, WS_2_-OH, and BT-OH at 800 °C are negligible, indicating excellent thermal stability. In contrast, the modified WS_2_@BT nanoparticles exhibit a relatively significant mass loss at temperatures above 400 °C, which is attributed to the thermal decomposition of the surface-grafted PEN-COOH. The TGA results confirm that PEN-COOH was successfully grafted onto the surfaces of WS_2_ and BT.

The XPS survey spectra of the nanofillers and the high-resolution spectra of the C1s and O1s elements are shown in Figure 2d–h. As illustrated in Figure 2f, the characteristic peaks of S2p and W4f (from WS_2_) as well as Ba3d, Ba4p, Ba4d, and Ti2p (from BT) are clearly observed in WS_2_@BT, confirming the coexistence of WS_2_ and BT in the nanohybrid. As shown in Figure 2g, The C1s fitted sub-peak spectra of WS_2_@BT show four diffraction peaks at 289.00 eV, 286.60 eV, 285.24 eV, and 284.58 eV, corresponding to O-C=O, C-O-C, C≡N, and C-C/C=C bonds, respectively [33,34,35,36]. Notably, the peaks at 289.00 eV and 286.60 eV originate from ester groups, indicating that the -OH groups on the BT-OH surface underwent an esterification reaction with the -COOH groups in PEN-COOH. As shown in Figure 2h, the O1s spectrum splits into two peaks at 530.81 eV and 529.91 eV, corresponding to Ti-O-Ti and Ba-O-Ti bonds [37], while additional split peaks at 533.66 eV, 532.83 eV, and 531.78 eV are attributed to C-O and C=O bonds. These results confirm the presence of PEN-COOH in WS_2_@BT. Collectively, the above characterization results demonstrate that PEN-COOH was successfully introduced into the surface of nanofillers.

Zeta potential is defined as the potential difference between the continuous phase and the fluid stabilization layer adsorbed on dispersed particles. Figure 2i shows the zeta potentials of the fillers in ultrapure water before and after modification. It can be seen that the zeta potential values of pure BT and WS_2_ are −11.7 mV and −10.5 mV, respectively, while those of WS_2_-OH and BT-OH are −13.7 mV and −28.5 mV, respectively. This is mainly attributed to the grafting of negatively charged -OH groups onto the surfaces of BT treated with hydrogen peroxide and WS_2_ treated with NaOH, which leads to a decrease in the zeta potential values of the hydroxylated BT and WS_2_. However, the Zeta potential of WS_2_@BT is −15.3 mV, which is due to the reaction between hydroxyl and carboxyl groups consuming part of the hydroxyl groups during the preparation of WS_2_@BT, resulting in a change in the Zeta potential of the WS_2_@BT nanofiller. This result further confirms the successful grafting of PEN-COOH onto the nanofiller surfaces.

**Figure 3:**
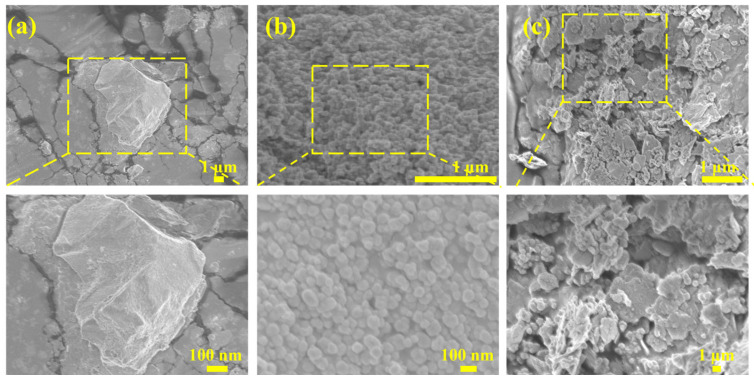
SEM images of functional nanofillers: (**a**) WS_2_; (**b**) BT; (**c**) WS_2_@BT.

To intuitively observe the microscopic morphology of the modified WS_2_@BT nanoparticles, pure WS_2_, BT, and WS_2_@BT nanofillers were examined using a scanning electron microscope (SEM), respectively. The test results are shown in Figure 3. As can be seen from Figure 3a, WS_2_ exhibits a flake-like morphology with a smooth surface. Figure 3b shows that the BT nanoparticles are spherical, with smooth surfaces and uniform sizes. For the WS_2_@BT nanofiller, Figure 3c shows that the spherical BT nanoparticles are successfully attached to the flake-like WS_2_ through PEN-COOH bridging, forming a novel three-dimensional nanofiller structure.

**Figure 4:**
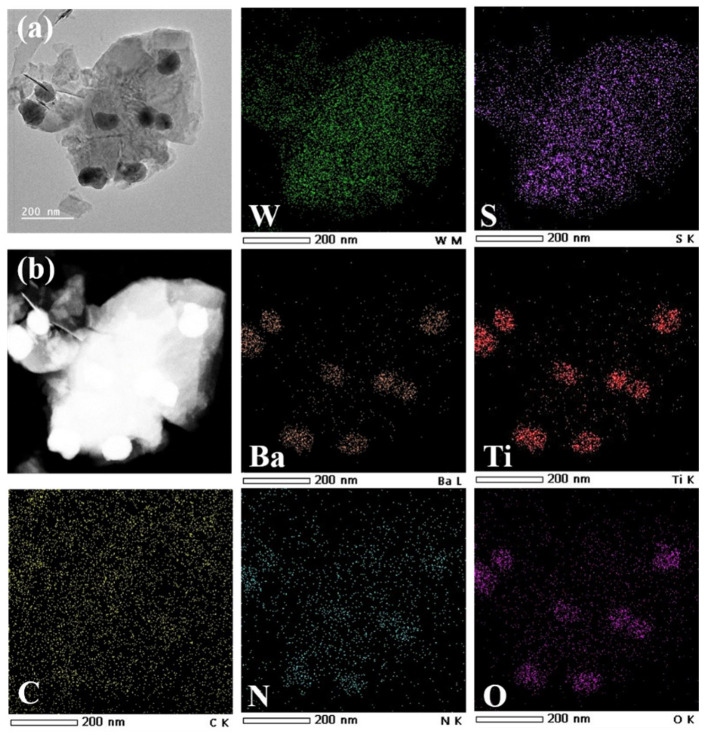
TEM images of functional nanofillers: (**a**) TEM of WS_2_@BT; (**b**) STEM of WS_2_@BT and TEM mapping images of WS_2_@BT.

To further observe the microscopic morphology with higher clarity on the basis of SEM characterization, transmission electron microscopy (TEM) analysis was conducted, and the TEM image of WS_2_@BT is shown in Figure 4. It can be clearly observed that multiple BT nanoparticles are closely loaded on the surface of the two-dimensional WS_2_ nanosheets. This is attributed to the formation of ester bonds between BT-OH, WS_2_-OH, and PEN-COOH, which interconnect to form a stable and novel three-dimensional structure. Meanwhile, TEM elemental mapping diagrams show the distribution of W and S elements on the WS_2_ nanosheets, while the distribution of Ba and Ti elements confirms the successful loading of BT nanoparticles. Additionally, the uniform distribution of C and N elements in the mapping diagrams verifies the successful grafting of PEN-COOH onto the nanoparticle surfaces.

**Figure 5:**
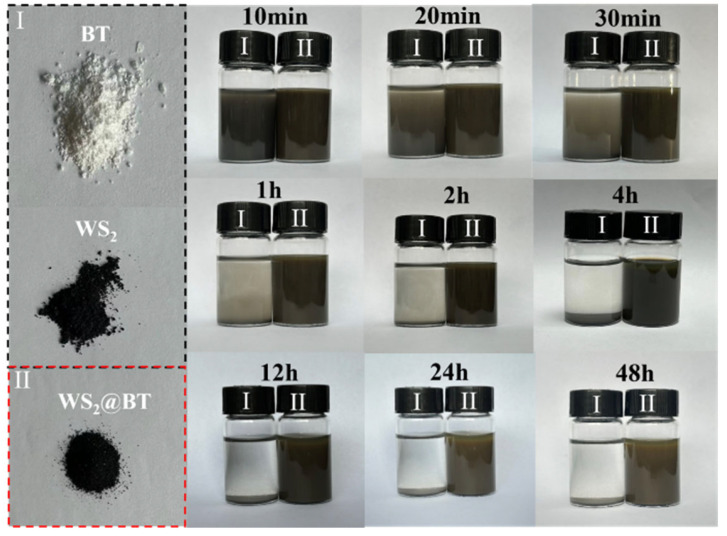
Images of the dispersion of nanofillers in NMP solvent before and after modification.

In addition to the aforementioned morphological and structural characterizations, the dispersibility of the fillers before and after modification was further evaluated, and the corresponding results are illustrated in Figure 5. Specifically, equal masses of unmodified and modified fillers were individually added to equal volumes of NMP solvent, followed by ultrasonic treatment to achieve homogeneous dispersion, after which the samples were allowed to stand for observation. After 30 min of standing, the solution containing unmodified WS_2_-BT exhibited obvious sedimentation, and a distinct delamination interface was observed after 1 h. In contrast, the modified WS_2_@BT nanofillers displayed excellent dispersibility, with no apparent sedimentation or delamination detected throughout the entire observation period. This superior dispersibility provides a robust foundation for the subsequent fabrication of high-performance composite materials.

Combined with the aforementioned characterization results from FT-IR, XRD, TGA, XPS, SEM, TEM, and dispersibility tests, it is further confirmed that hydroxylated WS_2_ and BT nanoparticles were successfully coated with PEN-COOH, and a novel 3D WS_2_@BT nanofiller was successfully prepared.

### 3.2. The Properties of Nanocomposites

**Figure 6:**
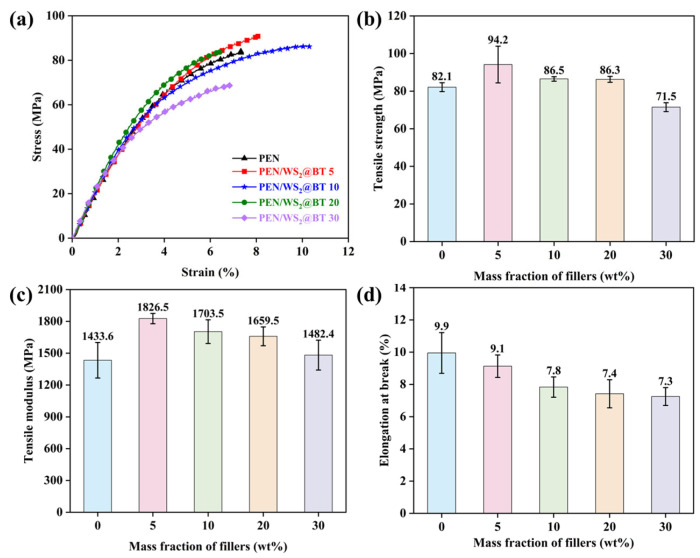
Mechanical properties of PEN/WS_2_@BT nanocomposite films: (**a**) stress–strain curves; (**b**) tensile strength; (**c**) tensile modulus; (**d**) elongation at break.

Mechanical properties are among the most critical indicators for evaluating the comprehensive performance of polymer composites. To systematically investigate the effects of nanofiller modification and filler loading on the mechanical behavior of PEN-based composites, a detailed study of the tensile strength, tensile modulus, and elongation at break was performed, with corresponding stress–strain curves presented in Figure 6.

As shown in Figure 6a,b, the tensile strength of the PEN/WS_2_@BT composite films initially increased and then gradually decreased with rising WS_2_@BT nanofiller content. The pure PEN film exhibited a tensile strength of 82.1 MPa, representing decent mechanical performance. When the WS_2_@BT loading reached 5 wt%, the tensile strength peaked at 94.2 MPa, corresponding to a 14.7% enhancement relative to PEN. This improvement is mainly attributed to the favorable interfacial compatibility between WS_2_@BT nanofillers and the PEN matrix, which promotes uniform dispersion and generates numerous internal stress-dissipation sites. Under an applied external load, stress can be efficiently redistributed across these sites, thereby elevating the tensile strength of the composites. Nevertheless, a further increase in WS_2_@BT content led to a gradual decline in tensile strength, which can be ascribed to excessive nanofiller aggregation caused by overloading, introducing local defects and deteriorating mechanical robustness [38]. Figure 6c displays the tensile modulus of the composites. All WS_2_@BT-filled samples exhibited a higher tensile modulus than pure PEN, which arises from the rigidifying effect of the inorganic nanofillers that restrict chain mobility and increase composite brittleness. As observed in Figure 6d, the elongation at break progressively decreased after the incorporation of WS_2_@BT nanofillers. This phenomenon results from the strong physical entanglement formed between WS_2_@BT and PEN macromolecular chains, which hinders the orientation and extension of PEN chains during tensile deformation. Despite the reduced elongation at break, the PEN/WS_2_@BT composites still possess outstanding overall mechanical properties and can satisfy the performance requirements for most practical applications.

**Figure 7:**
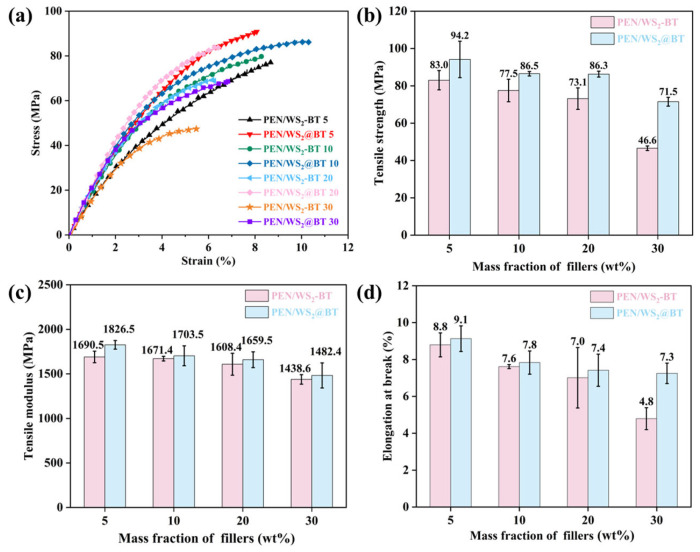
Mechanical properties of PEN nanocomposite films: (**a**) stress–strain curves; (**b**) tensile strength; (**c**) tensile modulus; (**d**) elongation at break.

Figure 7 compares the mechanical properties of PEN composite films filled with unmodified WS_2_-BT and modified WS_2_@BT nanofillers at identical loadings. As illustrated in Figure 7a,b, as the filler content increased from 5 wt% to 30 wt%, the tensile strength of PEN/WS_2_-BT films dropped sharply from 83.0 MPa to 44.6 MPa, whereas that of PEN/WS_2_@BT films decreased moderately from 94.2 MPa to 71.5 MPa. As further supported by Figure 7c,d, films filled with modified WS_2_@BT exhibited superior tensile modulus and elongation at break relative to their unmodified counterparts. The reinforcing efficiency of modified WS_2_@BT nanofillers is therefore markedly superior to that of unmodified WS_2_-BT. This can be explained by the uniform loading of BT nanoparticles onto the WS_2_ nanosheet surface, which increases surface roughness and specific surface area, thereby enlarging the contact area with the PEN matrix and strengthening physical entanglement and interfacial compatibility. Upon exposure to external stress, the well-compatibilized interface enables effective stress transfer and energy dissipation from the polymer matrix to the nanofillers, alleviating local stress concentration and enhancing the mechanical performance of the nanocomposite films.

**Figure 8:**
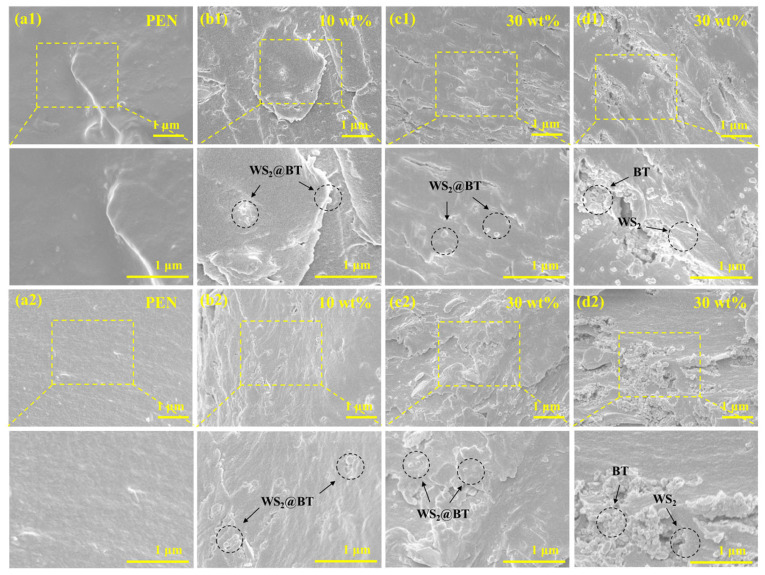
SEM images of PEN and CPEN-based nanocomposite films: (**a1**) PEN; (**b1**) PEN/WS_2_@BT 10; (**c1**) PEN/WS_2_@BT 30; (**d1**) PEN/WS_2_-BT 30; (**a2**) CPEN; (**b2**) CPEN/WS_2_@BT 10; (**c2**) CPEN/WS_2_@BT 30; (**d2**) CPEN/WS_2_-BT 30.

It also has been well established that the interfacial microstructure between nanofillers and the polymer matrix is a key factor determining the overall performances of polymer-based nanocomposites, encompassing thermal, mechanical, and dielectric properties. Since the dispersion and compatibility of nanofillers in the polymer matrix are directly related to the interfacial microstructure, these characteristics can be intuitively observed through scanning electron microscope (SEM) images [39]. The cross-sectional microstructures of the nanocomposites fabricated in this work are presented in Figure 8. As shown in Figure 8(a1), the cross-sectional morphology of the pure PEN film is relatively smooth and compact, representing the typical morphology of a pristine polymer film. The cross-sectional micrographs of the PEN/WS_2_@BT 10 and PEN/WS_2_@BT 30 composite films are displayed in Figure 8(b1) and Figure 8(c1), respectively. WS_2_ nanosheets decorated with BT nanoparticles are clearly observed to be uniformly dispersed within the PEN matrix. Furthermore, the surface grafting of PEN-COOH endows the nanofillers with excellent compatibility with the PEN matrix, as evidenced by the absence of obvious phase separation. However, for the PEN/WS_2_-BT 30 composite film filled with unmodified WS_2_ and BT nanoparticles (Figure 8(d1)), massive nanoparticle agglomerations and distinct phase separation between the fillers and the polymer matrix are observed, which validates the effectiveness of the surface modification strategy.

The cross-sectional morphologies of composite films after high-temperature heat treatment-induced solid-phase crosslinking are presented in Figure 8(a2–c2). No individually dispersed WS_2_@BT nanofillers are observed. Instead, the nanofillers are almost entirely and uniformly encapsulated by the crosslinked CPEN matrix, leading to further improved interfacial compatibility. In contrast, numerous agglomerated nanoparticles detached from the polymer matrix are still visible in Figure 8(d2). Collectively, these results demonstrate that the surface-modified nanofillers exhibit enhanced dispersion and interfacial compatibility in the polymer matrix, and high-temperature heat treatment can further improve the structural homogeneity of the composite films.

To systematically evaluate the influence of high-temperature heat treatment on the performance of PEN-based composites, the structure, thermal and electrical properties of PEN/WS_2_@BT and CPEN/WS_2_@BT composites before and after heat treatment were further investigated.

**Figure 9:**
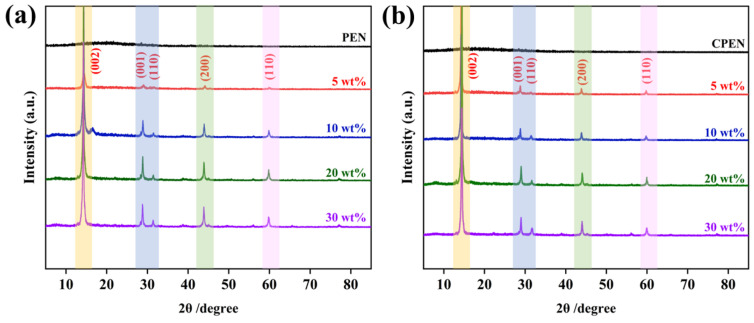
XRD patterns of nanocomposite films: (**a**) PEN/WS_2_@BT; (**b**) CPEN/WS_2_@BT.

Specifically, the crystal structure stability of the composites before and after heat treatment are shown in Figure 9. Pure PEN exhibits no obvious diffraction peaks, and as the WS_2_@BT loading increases, the intensity of the diffraction peaks gradually increases without any shift in their positions, indicating that the filler content does not alter the crystal structure of the PEN/WS_2_@BT composites. Furthermore, after high-temperature treatment, the position and intensity of the diffraction peaks remain nearly unchanged, which demonstrates that the crystal structures of WS_2_ and BT can be maintained intact after heat treatment.

**Figure 10:**
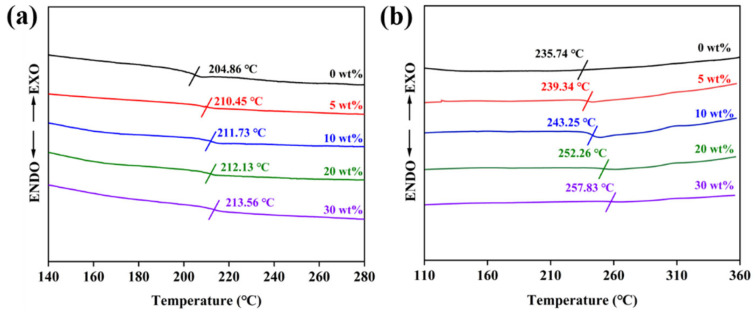
DSC curves of nanocomposite films: (**a**) PEN/WS_2_@BT; (**b**) CPEN/WS_2_@BT.

In addition, as shown in Figure 10, the glass transition temperature (*T*_g_) of the composite film, an important indicator reflecting its thermal stability, was analyzed through the DSC curve. For unheated samples, pure PEN has a *T*_g_ of 204.86 °C, and the *T*_g_ of the composite films gradually increases from 210.45 °C to 213.56 °C with the increase in WS_2_@BT content, which is attributed to the uniform dispersion of nanofillers that restricts the mobility of PEN molecular chains. For heat-treated samples, the CPEN composites show a further elevated *T*_g_, which is ascribed to the crosslinking reaction between the cyano groups on the CPEN chains and those on WS_2_@BT, forming a restrictive crosslinked network. These results collectively confirm the good high-temperature stability of both PEN and CPEN composites, laying a solid theoretical foundation for their practical application in high-temperature environments.

**Figure 11:**
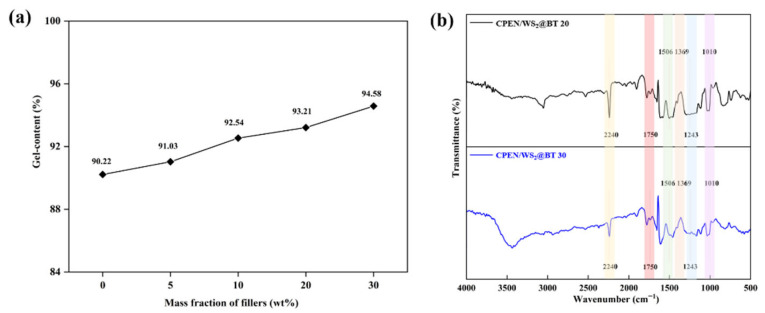
Gel-content (**a**) and FTIR spectra (**b**) of CPEN/WS_2_@BT nanocomposite films.

Since crosslinking degree is a key indicator of the performance of CPEN/WS_2_@BT composites, its characterization was also carried out, as shown in Figure 11a. The heat-treated composites exhibit a gel-content exceeding 90%, indicating high crosslinking efficiency. Specifically, the incorporation of inorganic nanofillers enhances the reaction density, promotes the activity of functional groups in the system, and increases the frequency of effective collisions, thereby improving the crosslinking degree and further optimizing the thermal properties of the composites. To further confirm the chemical structure evolution of CPEN composites after high-temperature treatment, FT-IR spectroscopy was used for verification, and the test results are shown in Figure 11b. An absorption peak at 1010 cm^−1^ corresponds to the phthalocyanine ring, while the characteristic peaks at 1506 cm^−1^ and 1369 cm^−1^ verify the formation of triazine rings [40]. Additionally, the strong absorption at 2240 cm^−1^ is assigned to the -CN groups in the PEN matrix [41], and the peak at 1750 cm^−1^ corresponds to the carbonyl groups in WS_2_@BT [42]. These results collectively confirm that triazine and phthalocyanine ring structures are successfully formed in the composites after high-temperature heat treatment.

**Figure 12:**
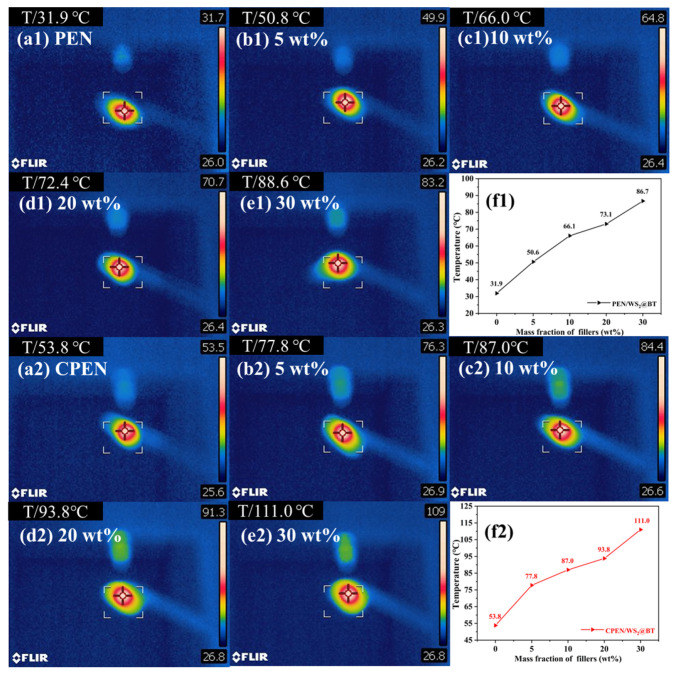
Infrared thermography images of PEN- and CPEN-based composite films with different filler contents: (**a1**–**e1**) PEN; (**a2**–**e2**) CPEN; (**f1**,**f2**) the center-temperature of nanocomposites.

In order to explore the heat transfer performance of PEN/WS_2_@BT and CPEN/WS_2_@BT composite films in practical applications, the films were irradiated with a laser for 10 s, and the surface temperature of the films was recorded using an infrared thermal imager (Figure 12). The surface temperature of pure PEN was only about 30 °C, while the surface center temperature of the PEN/WS_2_@BT composites gradually increased with the filler content, reaching 88.6 °C at 30% loading. After high-temperature treatment, the surface center temperature of CPEN composites is further enhanced, confirming the good thermal conductivity of the prepared composites. This excellent thermal conductivity endows the composites with potential application in electronic component thermal management.

**Figure 13:**
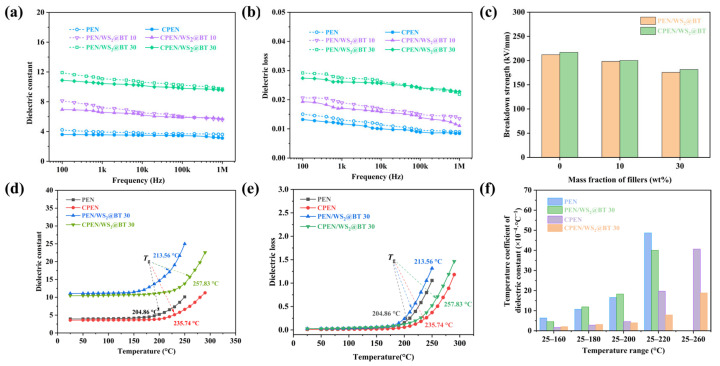
Dielectric properties of nanocomposite films: (**a**) dielectric constant; (**b**) dielectric loss; (**c**) breakdown strength; (**d**) dielectric constant-temperature relationship; (**e**) dielectric loss–temperature relationship; (**f**) dielectric constant-temperature coefficient.

Figure 13 presents the dielectric properties of PEN/WS_2_@BT and CPEN/WS_2_@BT composite films from 100 Hz to 1 MHz at room temperature. As illustrated in Figure 13a, the dielectric constant of all composite films decreases with the increase in frequency, while maintaining good frequency stability throughout the tested frequency range. Specifically, the dielectric constant of neat PEN at 1 kHz is merely 3.9, whereas with the increase in WS_2_@BT nanofiller content from 10 to 30 wt%, the dielectric constant of PEN/WS_2_@BT composites at 1 kHz increases to 7.2 and 11.1, respectively. After high-temperature treatment, the dielectric constant of CPEN/WS_2_@BT composites is slightly lower than that of corresponding PEN/WS_2_@BT composites under the same filler content. Compared with pure CPEN, the CPEN/WS_2_@BT composite with 30 wt% filler content achieves a 193% increase in dielectric constant. The enhancement of dielectric constant is mainly associated with two aspects: (1) both WS_2_ nanosheets and BT nanoparticles exhibit relatively high intrinsic dielectric constants, and their uniform dispersion within the PEN matrix contributes to the overall dielectric response of the composites. (2), the heterogeneous interfaces formed among WS_2_, BT, and the polymer matrix induce significant Maxwell–Wagner–Sillars (MWS) interfacial polarization under an external electric field, thereby markedly enhancing the dielectric constant [39].

In addition, Figure 13b shows the dielectric loss of PEN/WS_2_@BT and CPEN/WS_2_@BT composite films at room temperature. Although the dielectric loss of the composites increases slightly with the increase in WS_2_@BT content, it remains below 0.030 at 1 kHz for all composite samples. This phenomenon is mainly due to the good interfacial compatibility between WS_2_@BT nanofillers and the PEN matrix, which reduces the generation of interfacial defects and inhibits local charge accumulation at defect sites. Furthermore, the functional groups on the surface of WS_2_@BT nanofillers form chemical bonds with the nitrile groups in the PEN molecular chain, which further suppresses the interfacial polarization of the composite system and thus maintains the dielectric loss at a low level.

The breakdown strength of the nanocomposites is illustrated in Figure 13c. As shown in the figure, the pure PEN film exhibits a high breakdown strength exceeding 210 kV/mm, demonstrating excellent voltage-withstand performance. Although the breakdown strength of the nanocomposites gradually decreases with increasing filler content, the reduction is relatively mild. This is mainly due to the uniform dispersion of WS_2_@BT hybrid fillers and strong interfacial adhesion via ester bonds, which effectively reduce interfacial defects and inhibit the formation of electric field distortion centers. Notably, the breakdown strengths of CPEN-based nanocomposites show slightly higher than that of the PEN-based nanocomposites, which is attributed to the chemically crosslinked network that reinforces structural integrity and suppresses charge carrier migration. These results confirm that the synergistic effect of uniformly dispersed WS_2_@BT fillers and the crosslinked matrix ensures both high dielectric performance and robust breakdown resistance.

To further elucidate the temperature dependence of dielectric properties, the variation in dielectric constant with temperature is shown in Figure 13d. The dielectric constant remains relatively stable below the glass transition temperature (*T*_g_), indicating limited dipolar mobility within the rigid polymer matrix. This stability is mainly attributed to the rigid aromatic backbone of the PEN matrix, the crosslinked structure formed by nitrile groups, and the strong filler–matrix interactions, all of which effectively constrain polymer chain motion and restrict dipole orientation. Once the temperature exceeds *T*_g_, the dielectric constant increases rapidly due to enhanced segmental motion of polymer chains, which promotes dipole orientation and interfacial polarization under the alternating electric field.

The temperature dependence of dielectric loss is presented in Figure 13e, and its variation shows a trend similar to that of the dielectric constant. Below the *T*_g_, the dielectric loss remains at a low level, suggesting negligible energy dissipation. This is attributed to the restricted chain mobility imposed by the rigid PEN backbone and the chemically crosslinked network, which suppresses charge carrier migration and conduction loss. Moreover, the uniform dispersion of WS_2_@BT nanohybrids and the strong interfacial adhesion via esterification bonds minimize interfacial defects for charge accumulation. When the temperature exceeds *T*_g_, dielectric loss increases abruptly due to the intensified polymer segmental motion, which enhances dipole relaxation and orientation. Meanwhile, the reduced matrix viscosity at elevated temperatures promotes ionic conduction, further contributing to the increased dielectric loss [21].

As shown in Figure 13f, the dielectric constant-temperature coefficient of all composites, calculated according to the formula reported in the literature [39]. It can be seen that CPEN-based composites exhibit superior high-temperature dielectric stability compared to PEN-based composites. In particular, the dielectric constant-temperature coefficient of CPEN/WS_2_@BT 30 remains below 1 × 10^−3^ °C^−1^ in the temperature range of 25 to 220 °C, indicating excellent temperature stability of the dielectric constant within this range. Nevertheless, with the further increase in temperature beyond 220 °C, the dielectric constant-temperature coefficient of the composites increases significantly, which is closely related to the intense thermal motion of molecular chains and the destruction of the micro-capacitor network.

**Figure 14:**
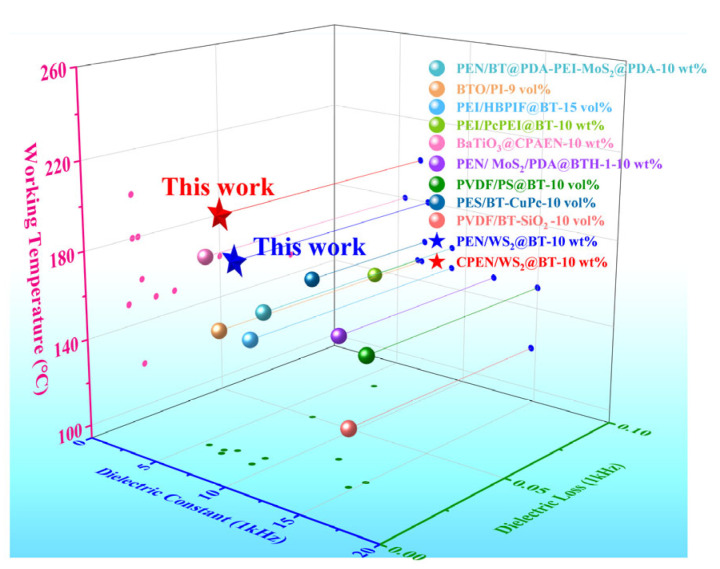
Comparison of dielectric properties of different composite dielectric materials [42,43,44,45,46,47,48,49,50].

Furthermore, Figure 14 summarizes the dielectric properties of various polymer-based composites filled with BT nanofillers reported in relevant studies. It is worth noting that many polymer composites, such as PVDF/PS@BT and PES/BT-CuPc composites, exhibit relatively high dielectric constants, but their service temperature is relatively low, usually below 150 °C, which greatly limits their application in high-temperature scenarios. In addition, some PEN-based nanocomposites incorporated with similar inorganic functional fillers, such as PEN/BT@PDA-PEI-MoS_2_@PDA, PEN/MoS_2_/PDA@BTH are also included in Figure 14. Although these nanocomposites have better temperature resistance than PVDF and PES-based composites, their dielectric properties are relatively inferior and their maximum service temperature is still lower than 160 °C. In contrast, the PEN/WS_2_@BT and CPEN/WS_2_@BT nanocomposites prepared in this work not only maintain excellent dielectric properties but also exhibit superior high-temperature resistance. Particularly, the service temperature of CPEN/WS_2_@BT composites exceeds 180 °C. This outstanding high-temperature resistance endows the composites with distinct advantages in practical applications, ensuring their adaptability to harsh high-temperature working conditions.

## 4. Conclusions

In conclusion, this study proposes a feasible method for preparing PEN-based nanocomposite films with excellent heat resistance, which are suitable for application in high-temperature environments. A novel structured nanofiller (WS_2_@BT) with good dispersibility was successfully synthesized by firmly attaching BT nanoparticles to WS_2_ nanosheets. Subsequently, the WS_2_@BT nanofillers were added to the PEN matrix as reinforcing and functional fillers, and the initial PEN/WS_2_@BT nanocomposites were prepared by a simple fabrication process. Furthermore, crosslinked CPEN/WS_2_@BT composites were further obtained via high-temperature heat treatment. The results demonstrate that the introduction of WS_2_@BT nanofillers can significantly enhance the comprehensive properties of PEN-based nanocomposite films. Specifically, when the filler loading is 5 wt%, the tensile strength and tensile modulus of the PEN/WS_2_@BT films are increased by 14% and 27% respectively compared with pure PEN. In addition, when the filler loading is 30 wt%, the CPEN/WS_2_@BT composites achieve a *T*_g_ of 257.83 °C, and their dielectric constant is 193% higher than that of pure CPEN. Most notably, the dielectric constant-temperature coefficient of the CPEN/WS_2_@BT composites remains below 1 × 10^−3^ °C^−1^ in the temperature range of 25–220 °C, which indicates excellent thermal and dielectric stability of the PEN-based nanocomposites. Therefore, these nanocomposites are believed to have broad application prospects in the field of high-temperature-resistant electronic devices.

## Data Availability

The original contributions presented in this study are included in the article. Further inquiries can be directed to the corresponding author.
